# An Interactive Module to Enhance Clinical and Non-clinical Communication Skills With LGBTQIA2S+ Patients

**DOI:** 10.7759/cureus.44999

**Published:** 2023-09-10

**Authors:** Michael K Nguyen, Sambina P Anthony, Matthew P Manganiello, Karla A Bell, Dimitrios Papanagnou

**Affiliations:** 1 Emergency Medicine, Thomas Jefferson University, Philadelphia, USA; 2 Population Health/Physical Therapy, Jefferson College of Rehabilitation Sciences, Thomas Jefferson University, Philadelphia, USA

**Keywords:** queer, diversity and inclusion, language and communication, training module, medical education, lgbtq+, communication training

## Abstract

The Lesbian, Gay, Transgender, Queer, Intersex, Asexual, Two-Spirit, and all others (LGBTQIA2S+) community comprises a diverse array of people who challenge conventional norms regarding sexual orientation and/or gender identity. This group possesses a distinct set of social and cultural principles that necessitate personalized and all-encompassing medical attention. In light of the increase in the number of individuals openly sharing their LGBTQIA2S+ identity and a growing societal openness toward this community, most healthcare providers do not feel prepared with the requisite knowledge and skills to appropriately care for the needs of this community. We describe the development of an educational intervention, the LGBTQIA2S+ Healthcare Module, to address this significant gap in health professions education. It offers current and future clinicians just-in-time training on the language and cultural context to adequately provide patient-centered care to this community.

## Editorial

The number of individuals in the United States who self-identify as Lesbian, Gay, Transgender, Queer, Intersex, Asexual, Two-Spirit, and all others (LGBTQIA2S+) is 7.2%, having doubled in the last decade, most likely due to a cultural attitude shift and growing social tolerance of the LGBTQIA2S+ community [1]. There is higher LGBTQIA2S+ identification with each younger generation as it is becoming safer for individuals to come out. However, individuals identifying as LGBTQIA2S+ continue to report lower general self-acceptance than heterosexual people, resulting in poorer mental health outcomes, including, depression symptoms and lower psychological well-being [2]. Although members of the LGBTQIA2S+ community experience discrimination in all parts of the world, those living in rural areas or countries with discriminatory legislation may be particularly vulnerable to stigma, social isolation, and risk of violence [3]. Because of these aforementioned implications, there is a growing need and push to integrate LGBTQIA2S+ health into the formal curriculum in standard medical education curricula. In this editorial, we not only describe this pressing need but also share a curricular resource with the medical education community that may be adopted at other institutions.

Current literature has demonstrated that LGBTQIA2S+ individuals have worse healthcare experiences and outcomes compared to straight and/or cisgender individuals [4]. They experience higher rates of anal cancer, asthma, cardiovascular disease, obesity, substance use, cigarette smoking, and suicide [5]. Members of the LGBTQIA2S+ community are less likely to have health insurance, have a regular primary care physician, and fill their prescriptions. They are more likely to experience delays in receiving care, refusal of healthcare services, and harassment by healthcare providers [6]. These disparities highlight the importance of integrating LGBTQIA2S+ sensitivity and health education into the training of physicians across all specialties and practices.

Presently, there is dissatisfaction from the medical community about the level of LGBTQIA2S+ topics in medical education. A 2011 study in JAMA by Obedin-Maliver et al. concluded that the deans of the United States and Canadian medical schools endorsed dissatisfaction with their institutions’ coverage of LGBTQIA2S+-related topics [7]. In a 2021 study, Canadian emergency medicine (EM) physicians felt that LGBTQIA2S+ individuals deserve equitable care when compared to cis-gender and heterosexual patients. However, they did not feel adequately prepared to care for these patients with the medical training they received [8]. Medical students reported a deficit in the amount of LGBTQIA2S+ health training they received and expressed interest in more robust educational opportunities [9]. Most students desire teaching to be spread throughout the curriculum and delivered by members of the LGBTQIA2S+ community via an interactive platform [9].

**Figure 1:**
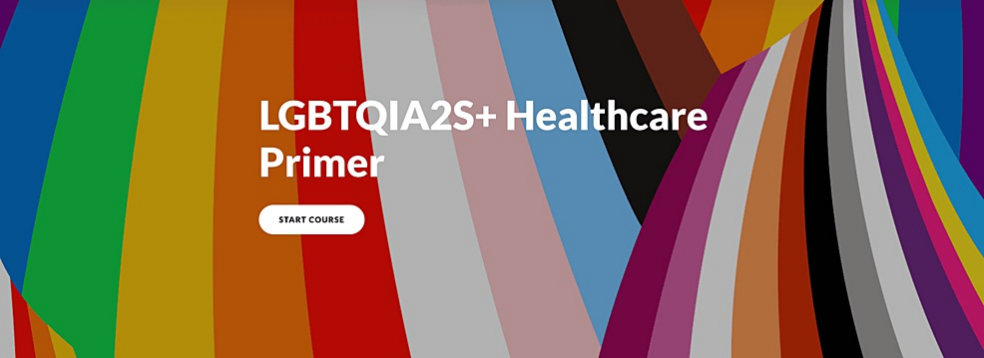
LGBTQIA2S+ healthcare primer

With the paucity of sexual orientation and gender identity training resources available to the medical education community, educators may encounter challenges when trying to incorporate LGBTQIA2S+ health into the medical education curriculum. After identifying this resource gap, we, as students of the Sidney Kimmel Medical College (SKMC) at Thomas Jefferson University in Philadelphia, PA, USA, developed an interactive module on LGBTQIA2S+ Health Care (Figure 1), which we describe in the paragraph below [10]. The purpose of this module is to educate healthcare professionals and trainees on effective communication and more sensitive care towards the LGBTQIA2S+ community. (Please note: this module is open access and is readily available through the link provided under reference #10 of this editorial.)

Our module, meticulously developed through a comprehensive literature review, addresses a gap in the literature and local curricular needs at our medical school. To ensure relevance, we incorporated student feedback, focusing on the intersection of patient-centered communication and language, clinical care, and LGBTQIA2S+ health. By harnessing the interactive capabilities of Articulate Rise, we enriched the module with engaging activities while adhering to best practices in instructional design. Alongside its concise format and alignment with our medical education program objectives, the module is well-suited for assigned readings. Featuring chapters such as “Why Language Matters,” “Pronoun Use,” “Gender and Anatomical Language,” “Taking a Sexual History,” “Correcting Language Mistakes,” “HIV Language and Stigma,” “Navigating Patient Discrimination,” “OB/GYN Language,” and “Reproductive History,” the module encompasses a comprehensive spectrum of LGBTQIA2S+ health education. We further enhanced the learning experience with interactive patient scenarios, self-reflection exercises, and mini games that foster active content absorption. Rigorous evaluation by a content expert at our institution (KB) ensured the module's accuracy and user-friendly nature.

Each chapter of the module includes specific learning objectives that coincide with a major LBGTQIA2S+ topic (e.g., gender and anatomic language). Chapters of the module that align with pre-clinical SKMC curricular learning objectives are provided to students as an asynchronous resource to complement their independent study. The module now represents a resource for case-based learning in the medical school curriculum and provides students with a more contextual understanding of LGBTQIA2S+ health. Preliminary feedback from students and faculty educators has been overwhelmingly positive. We intend to aggregate student satisfaction data, as well as student outcomes on block examinations, at the end of the academic year.

In summary, significant health disparities stem from a lack of LGBTQIA2S+ health integration into most medical school curricula. It is our hope that this module will provide medical school students and faculty with the skills necessary to deliver equitable care to LGBTQIA2S+ patients. We invite other educators to consider its content and/or adoption in their respective curricula. We intend to continue to offer this module to both faculty and students in order to evaluate its effectiveness. These findings may have a meaningful clinical impact on medical education curricula and LGBTQIA2S+ care, especially as the LGBTQIA2S+ community continues to grow in the United States.
